# Reconstructed evolutionary patterns for crocodile-line archosaurs demonstrate impact of failure to log-transform body size data

**DOI:** 10.1038/s42003-022-03071-y

**Published:** 2022-02-25

**Authors:** Roger B. J. Benson, Pedro Godoy, Mario Bronzati, Richard J. Butler, William Gearty

**Affiliations:** 1 Department of Earth Sciences, University of Oxford, Oxford, OX1 3AN UK; 2 Departamento de Zoologia, Universidade Federal do Paraná, 81531–980 Curitiba, Paraná Brazil; 3 Department of Anatomical Sciences, Stony Brook University, Stony Brook, NY USA; 4 Department of Biology, University of Sao Paulo, 13840-091 Ribeirao Preto, SP Brazil; 5 School of Geography, Earth & Environmental Sciences, University of Birmingham, Birmingham, B15 2TT UK; 6 School of Biological Sciences, University of Nebraska-Lincoln, Lincoln, NE USA

**Keywords:** Phylogenetics, Palaeontology

**arising from** M.T. Stockdale and M.J. Benton. *Communications Biology* 10.1038/s42003-020-01561-5 (2021)

Pseudosuchia includes crocodylians, plus all extinct species more closely related to them than to birds. They appeared around 250 million years ago and have a rich fossil history, with extinct diversity exceeding that of their living members^1–3^. Recently, Stockdale & Benton^4^ presented analyses of a new dataset of body size estimates spanning the entire evolutionary history of Pseudosuchia. They quantified patterns of average body size, body size disparity through time and rates of evolution along phylogenetic lineages. Their results suggest that pseudosuchians exhibited considerable variation in rates of body size evolution, for which they provided various group-specific explanations and asserted the importance of climatic drivers. This differs from two recent studies that analysed a substantial portion of pseudosuchian body size evolution and proposed that adaptation to aquatic life, a biological innovation of some subgroups, was the main driver of body size evolution, with patterns of disparity also being influenced by size-dependent extinction risk^5,6^. Here we show that the analytical results of Stockdale & Benton^4^ are strongly influenced by a methodological error in their body size index. Specifically, that they chose not to log-transform measurement data prior to analyses.

Stockdale & Benton^4^ recorded 21 measurements across 280 species. Most of their measurements (17) were cranial and four were from the limb skeleton. They submitted these measurements to a principal components analysis (PCA) and iterative missing data estimation procedure^7^, then used principal component 1 (PC1) scores as a size index. This method was intended to overcome the problem of using skull length on its own as a size proxy, which may be biased by variation in relative head size and snout length^4^. Indeed, Stockdale & Benton^4^ suggested that this bias might explain key differences between their findings and the results of the previous studies^5,6^. Nevertheless, their PC1 size index is highly correlated to skull length (Fig. 1 and see below; regardless of data treatment), and one previous study did also address the biasing effects of snout length variation by excluding the snout from skull length measurements^5^. Therefore, we did not expect such strongly different results on that basis alone. Instead, we argue that key differences result mainly from the fact that previous studies used log-transformed size indices^5,6^, whereas Stockdale & Benton^4^ did not.

**Fig. 1:**
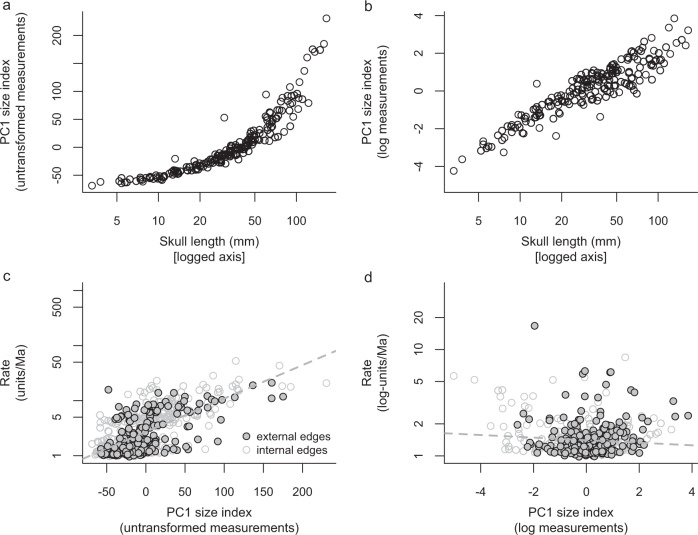
Effects of log-transformation on the PC1 size index of Stockdale & Benton (2021). **a** Original (untransformed) version of the PC1 size index shows a curved relationship with log-transformed skull length (Spearman’s ρ = 0.98, *p* < 0.00001, *N* = 202; using rank-based correlation due to the curved nature of the relationship). **b** Log-transformed version of the PC1 size index shows a linear relationship with log-transformed skull length (see text for correlation test results). **c** Evolutionary rates estimated from the original (untransformed) version of the PC1 size index are not independent of size. **d** Evolutionary rates estimated from the log-transformed version of the PC1 size index are independent of size.

Measurements of real-world objects are taken in additive (or absolute) units such as millimetres (mm). This presents a well-understood statistical problem because biological variation is often multiplicative (=relative, or proportional), such that variance increases with increasing scale (heteroskedasticity; e.g. refs. ^8,9^). For example, a hypothetical group of rodents has species-mean body masses ranging from 100 to 200 grams, whereas hypothetical artiodactyls range from 100 to 200 kg. Both groups exhibit identical (two-fold) relative variation, but this is not reflected when additive units are analysed because the absolute difference between the largest and smallest artiodactyls is 1000 times that in rodents. Therefore, size variation among large-bodied species is substantially over-weighted unless measurements are log-transformed (see refs. ^8,9^). To ignore this, either by analysing or simulating trait data in a non-logged context, is to model a version of evolution in which it is as easy for a mouse population to evolve a body size increase of 10 kg as it is for an elephant population.

When using log units instead of additive units, identical proportional increases are indexed by identical numerical increases, entirely solving the problem (e.g. our rodents span from 2.0 to 2.3 log_10_-grams, cf. 5.0–5.3 log_10_-grams in artiodactyls). Therefore, evolutionary rate studies have routinely used log-transformed measurements for more than 70 years^8–10^. This is especially important when measurements span across orders of magnitude^9^, as with pseudosuchians which range from the largest species, *Sarcosuchus imperator* (skull length = 1650 mm) to the smallest, *Knoetschkesuchus guimarotae* (33 mm). This represents an estimated 125,000-fold variation in body mass, approximating that body mass scales with the cube of linear dimensions (or 15,000-fold, conservatively reducing the skull of *Sarcosuchus* by 50% to account for its proportionally long snout). Although we illustrate the nature of this problem using extremes, it cannot be addressed just by excluding small-and large-bodied taxa from analyses, because variance increases as a continuous function of scale.

We replicated the analyses of Stockdale & Benton^4^, using log_10_-tranformed measurements. This resulted in a modified version of their PC1 size index that scales linearly with, and is strongly correlated to, log-transformed skull length (Fig. 1b; *p* < 0.001; R^2^ = 0.85; *N* = 202; Pearson’s product-moment). In contrast, the non-logged version^4^ shows a curved relationship (Fig. 1a) that upweights relative variation among large-bodied species—~80% of the variation in their index (*y*-axis) represents less than 25% of variation in relative (log-transformed) size. Due to space limitations, we focus only on macroevolutionary model comparisons and variation in evolutionary rates mapped to phylogeny. These analyses are central to the conclusions of Stockdale & Benton^4^ because they document variation in the tempo and mode of evolution among groups of different ages. Therefore, they provide process-based explanations that underpin the interpretation of their other analytical outputs, such as patterns of variation in disparity and average rates through time.

Re-analysis of rate variation among phylogenetic lineages^11^ using the non-logged PC1 size index returns similar results to those of Stockdale & Benton^4^ (Fig. 2a). Evolutionary rates returned by this analysis are measured in additive (or absolute) length units per million years and high rates occur in two contexts: (1) On the lineages leading to some large-bodied species such as the notosuchian *Razanandrongobe*, the phytosaur *Angistorhinus*, and the early crocodylomorph *Carnufex*; and (2) In some clades or grades of generally large-bodied species, including various Triassic pseudosuchians, Tethysuchia, Thalattosuchia and some eusuchians. However, these instances of high evolutionary rates are artefacts resulting from an underlying correlation of high rates with large body sizes (Fig. 1c; R^2^ = 0.21; *p* < 0.001; *N* = 281 species; phylogenetic least squares regression [PGLS]; R^2^ = 0.46; *p* < 0.001; *N* = 559 species and nodes; ordinary least squares regression [OLS]). This is expected when data are not log-transformed because using non-logged measurements inflates the amount of evolutionary change inferred to have occurred among large-bodied species.

**Fig. 2:**
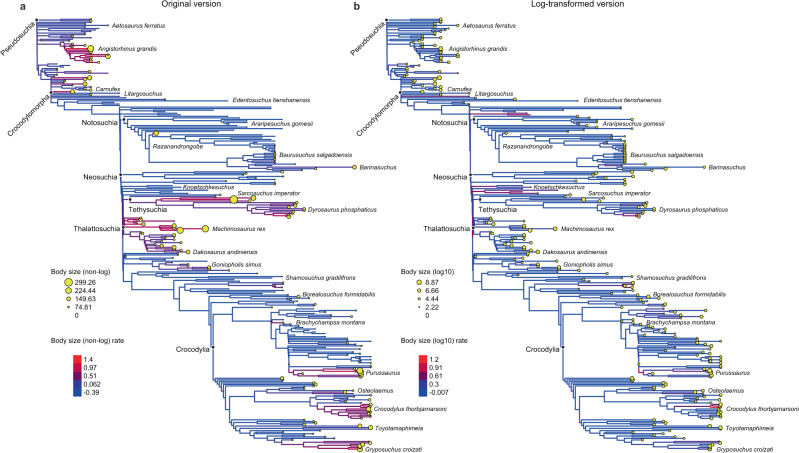
Phylogenetic patterns of rate variation inferred from original and log-transformed data showing highly different results. Rate variation mapped to phylogeny using colours based on **a** the original (untransformed) version of the PC1 size index, compared to those from **b** the log-transformed version of the PC1 size index. Yellow circles at the tips of the tree are scaled according to species body size.

Analysis of our log-transformed version of the PC1 size index documents rate variation in relative length units per million years and is uncorrelated or very weakly correlated with variation in absolute size (Fig. 1d; R^2^ = 0.03; *p* = 0.004; *N* = 281 species; PGLS; R^2^ = 0.008; *p* = 0.19; *N* = 559; OLS). This rate variation shows a very different pattern to that of Stockdale & Benton^4^ (Fig. 2b), rejecting the occurrence of high evolutionary rates in large-bodied groups and species, including those listed above. This removes the need for hypotheses such as early evolutionary radiation of Triassic pseudosuchians, island endemism in *Razanandrongobe*, and viviparity in thalattosuchians^4^. These processes may well be important drivers of phenotypic evolution and species diversification in those taxa, but they did not result in above-background rates of body size evolution. Instead, we find a much more even distribution of high rates through time and among large- and small-bodied lineages, including high rates involved in the attainment of small body size in groups such as atoposaurids and shartegosuchids.

Nevertheless, biological interpretation of these patterns should be avoided because we find no support for the variable-rate model compared to a uniform-rate Brownian motion (BM) model (marginal likelihood = –477.2 compared to −470.7 for BM). Therefore, the variable rates model may be over-parameterised and should not be interpreted closely, overturning the conclusions of Stockdale & Benton^4^, including their time series of rate variation, which is based on this model. We also find strong support for a constrained, Ornstein-Uhlenbeck (OU), model compared to BM (marginal likelihood_OU_ = −463.5), consistent with previous studies^5,6^, but differing from Stockdale & Benton^4^. The constraint parameter of the OU model (α) is estimated as 0.016. This corresponds to a phylogenetic half life^12^ of 43 million years, which is short compared to the study duration of ~250 million years. Pseudosuchian body size evolution is therefore highly distinguishable from Brownian (diffusive) evolution, consistent with the importance of functional and ecological limits to body size evolution at large phylogenetic scales^5,6^. A speciational (kappa) model is best supported (marginal likelihood_kappa_ = −454.0), but we disagree with the interpretation^4^ that this provides evidence of punctuational evolution, given that only a small proportion of species that ever lived are actually sampled in the fossil record.

Our analyses of log-transformed data reject key aspects of the conclusions of Stockdale & Benton^4^. More broadly, they demonstrate that the decision not to log-transform measurements introduces substantial errors to inferences of variation in the rate of evolution, and this should be accounted for in future studies.

## Methods

We used the measurements provided by Stockdale & Benton^4^ to reproduce their published analyses of rate variation, also analysing a version in which the input data were log-transformed prior to analysis. Principal component analysis (PCA) with iterative missing data imputation was carried out in PAST version 3.1^7^. We used the scores of the first principal component (PC1) as a body size index. Rate variation was evaluated on the time-scaled phylogeny of Stockdale & Benton^4^, using the ‘varRates’ model of BayesTraits version 3.0.2^11^ and running our mcmc analyses for 2 million generations, with 50% of these discarded as burn-in (compared to 2 million generations with 10,000 discarded as burn-in by Stockdale & Benton^4^). Results were summarised using tools available at www.evolution.reading.ac.uk/VarRatesWebPP. We used mean scalar values as an estimate of evolutionary rates following Stockdale & Benton^4^, but our scripts allow the use of alternative rate summary metrics (Supplementary data).

Phylogenetic rate variation was visualised by painting rate colours to phylogenetic branches using functions from the R package ape version 5.0^13^, in R version 4.0.3^14^. We also used functions from ape to extract estimated body sizes at internal nodes of the phylogeny and to graphically compare body size to evolutionary rates (Fig. 2). We statistically tested the correlation of evolutionary rates to body size using ordinary least squares regression of node- and tip-rates to body size, and also using phylogenetic generalised least squares regression (PGLS) to compare summed root-to-tip rates to the PC1 body size index, following e.g. ref. ^15^. This was implemented using custom code (Supplementary data) and the pgls function of the R package caper version 1.0.1^16^.

Finally, to evaluate support for a variable rates model compared to alternatives, we conducted a model comparison analysis in BayesTraits^11^. This was based on stepping-stone sampling, running 10 stones each for 100,000 generations following 1,000,000 generations of burn-in, comparing varRates to speciational (kappa), Ornstein-Uhlenbeck and Brownian motion models.

## Data Availability

The datasets generated and analysed during the current study are available in the Figshare repository 10.6084/m9.figshare.15147306. This includes measurements from the supplementary data of Stockdale & Benton^4^, as well as other data required to replicate our analyses.

## Code availability

The analytical scripts used in the current study are available in the Figshare repository^17^.
